# Mangrove Habitat Use by Juvenile Reef Fish: Meta-Analysis Reveals that Tidal Regime Matters More than Biogeographic Region

**DOI:** 10.1371/journal.pone.0114715

**Published:** 2014-12-31

**Authors:** Mathias M. Igulu, Ivan Nagelkerken, Martijn Dorenbosch, Monique G. G. Grol, Alastair R. Harborne, Ismael A. Kimirei, Peter J. Mumby, Andrew D. Olds, Yunus D. Mgaya

**Affiliations:** 1 Radboud University Nijmegen, Institute for Water and Wetland Research, Department of Animal Ecology and Ecophysiology, Nijmegen, The Netherlands; 2 Tanzania Fisheries Research Institute, Dar es Salaam, Tanzania; 3 Southern Seas Ecology Laboratories, School of Biological Sciences and The Environment Institute, The University of Adelaide, Adelaide, Australia; 4 Marine Spatial Ecology Laboratory and Australian Research Council Centre of Excellence for Coral Reef Studies, School of Biological Sciences, The University of Queensland, Brisbane, Australia; 5 Tanzania Fisheries Research Institute-Kigoma Center, Kigoma, Tanzania; 6 Australian Rivers Institute – Coast and Estuaries and School of Environment, Griffith University, Gold Coast, Australia; 7 College of Natural and Applied Sciences, Department of Aquatic Science and Fisheries, University of Dar es Salaam, Dar es Salaam, Tanzania; University of Otago, New Zealand

## Abstract

Identification of critical life-stage habitats is key to successful conservation efforts. Juveniles of some species show great flexibility in habitat use while other species rely heavily on a restricted number of juvenile habitats for protection and food. Considering the rapid degradation of coastal marine habitats worldwide, it is important to evaluate which species are more susceptible to loss of juvenile nursery habitats and how this differs across large biogeographic regions. Here we used a meta-analysis approach to investigate habitat use by juvenile reef fish species in tropical coastal ecosystems across the globe. Densities of juvenile fish species were compared among mangrove, seagrass and coral reef habitats. In the Caribbean, the majority of species showed significantly higher juvenile densities in mangroves as compared to seagrass beds and coral reefs, while for the Indo-Pacific region seagrass beds harbored the highest overall densities. Further analysis indicated that differences in tidal amplitude, irrespective of biogeographic region, appeared to be the major driver for this phenomenon. In addition, juvenile reef fish use of mangroves increased with increasing water salinity. In the Caribbean, species of specific families (e.g. Lutjanidae, Haemulidae) showed a higher reliance on mangroves or seagrass beds as juvenile habitats than other species, whereas in the Indo-Pacific family-specific trends of juvenile habitat utilization were less apparent. The findings of this study highlight the importance of incorporating region-specific tidal inundation regimes into marine spatial conservation planning and ecosystem based management. Furthermore, the significant role of water salinity and tidal access as drivers of mangrove fish habitat use implies that changes in seawater level and rainfall due to climate change may have important effects on how juvenile reef fish use nearshore seascapes in the future.

## Introduction

Coastal habitats play an important role as nurseries in the early life history of many marine fish species. Juvenile and adult habitats of various marine fish species are spatially separated, and habitats where juveniles spend most of their life are often referred to as nursery habitats [1], [2]. The use of the term “nursery habitat” has received considerable attention [1]–[4]. The general consensus is that a particular habitat can be referred as a nursery habitat if it contributes a higher than average biomass to a spatially separated adult population compared to all other juvenile habitats. This can be realized through enhanced fish density, growth, survival, or movement to adult habitats of juveniles in nursery habitats compared to other nearby habitats [2]. Others have suggested a broader application of the concept by using the overall contribution of nursery habitats to adult populations rather than the contribution per unit area [1]. However, the most important parameter is the functional movement of fish from juvenile to adult habitats. Most coral reef fish species are known to have two life stages: a pelagic larval stage and a demersal juvenile and adult stage [5]. Juveniles of many fish species do not, however, settle directly in adult habitats after having completed their pelagic larval phase, but instead undertake ontogenetic habitat shifts during which they move across a variety of shallow-water habitats [3], [6].

Several habitats, such as mangroves, seagrass beds, patch reefs, mudflats, salt marshes, estuaries and associated habitats, act as nursery grounds for juveniles of numerous fish species [7]–[9]. Their nursery role is underpinned by the provisioning of resources, such as food or shelter, for many invertebrates and fish species [10], [11]. Most evidence based on stable isotope analyses suggests that mangrove habitats are predominantly used for shelter while seagrass beds perform an important role as feeding grounds [12]–[14]. The absence of such habitats is correlated with a significantly lower density and diversity of adults on nearby coral reefs [4], [15], [16], as well as lower rates of key ecological processes on reefs [17], [18]. Therefore, coastal habitats do not function as isolated entities, but rather are connected to each other by tides and fish movements. However, each habitat may function differently depending on its position in the seascape [7], [19]. Apart from functioning as nursery habitat, these habitats are also known to energetically subsidize one another through carbon fluxes and exchange of other materials, although the extent and magnitude of carbon exchange form the basis of an ongoing debate [14], [20], [21].

The nursery value of shallow-water tropical ecosystems depends partly on habitat accessibility to juvenile fishes [6], [22]. While habitat accessibility to fishes is high in regions such as the Caribbean due to the permanent inundation of coastal vegetated habitats (especially mangroves), similar habitats can only be accessed at high tide in large parts of the Indo-Pacific region that are influenced on a daily basis by large tidal amplitudes [22]–[24]. Tidal changes in the Indo-Pacific region are important in structuring the fish fauna in shallow water habitats and determine connectivity with other intertidal and subtidal habitats [22], [24], [25]. Tidal regime and seascape structure can profoundly influence habitat connectivity thereby affecting the fish assemblages in and the value of, marine reserves [24], [26]–[29]. Also salinity has been reported to structure fish assemblages, especially in estuarine environments, where the upper, middle, and lower estuaries often harbor different fish assemblages [30], [31]. Fish species vary in their salinity tolerances and preferences [32]. Therefore, fish communities are often structured in accordance with local salinity gradients [30], [33], [34].

With the steadily increasing number of studies that have focused on nursery function, the role of mangroves, salt marshes, and seagrass beds as habitats for aquatic organisms has been the subject of several reviews [4], [23] and meta-analyses [8], [10], [35]. Meta-analysis has the advantage over reviews that it can calculate and statistically test an effect size across a multitude of studies for the variable in consideration. Separate meta-analyses have examined the role of temperate seagrass [10], mangroves [35] and salt marsh [8] as nurseries for juvenile fish and decapod crustaceans. These meta-analyses concluded that abundance, growth and survival of animals were significantly higher in vegetated than in un-vegetated habitats. However, such analyses have typically focused on single habitats, which have limited larger seascape-scale insights into habitat usage by nursery species at different life stages or across different trophic levels [36]. Furthermore, in most studies juvenile and adult densities are pooled together [37], [38]. Hence, there is a gap in understanding of large-scale patterns in the use of multiple, connected shallow coastal habitats by juvenile fish, and underlying environmental drivers such as tide which has been postulated to potentially act as a major driver of nursery habitat use [29], [39]. We here test the hypothesis that differences in tidal amplitude and salinity across regions play a major role in determining habitat use by fishes during their early life stages. We performed a meta-analysis that examined the usage of various tropical coastal habitats (seagrass beds, coral reef and mangroves) by juvenile reef fishes on a global scale. The result is a large-scale overview of the ecological functioning of, and potential ecological connectivity among, habitats in tropical coastal ecosystems. This not only provides us with a better understanding of fish habitat use in the wider seascape and the unique role that some habitats may play, but also sheds light on the potential underlying mechanisms that lead to differences in ecological connectivity across habitats, with important implications for fishes, fisheries and ecosystem functioning.

## Material and Methods

### Literature search and database construction

We searched the literature using Thomson Reuters' Web of Knowledge and Elsevier's Science Direct electronic databases using the following keywords as topics: 1) a combination of mangroves* and seagrass*, together with ‘fish’, ‘fisheries’, ‘decapods’, ‘crabs’, ‘prawns’ or ‘juveniles’; 2) a combination of mangroves* and seagrass*, together with ‘growth’, ‘biomass’, ‘density’ or ‘survival’. The results were thereafter filtered using ‘nursery’ as a keyword to narrow the output down into relevant studies before carefully examining the methods and the species studied therein. In addition, we searched the reference lists of several review papers [3], [4], [6], [10], [23], [35], [36] and included relevant unpublished data of our own (S1 Fig. & S1 Table).

**Figure 1:**
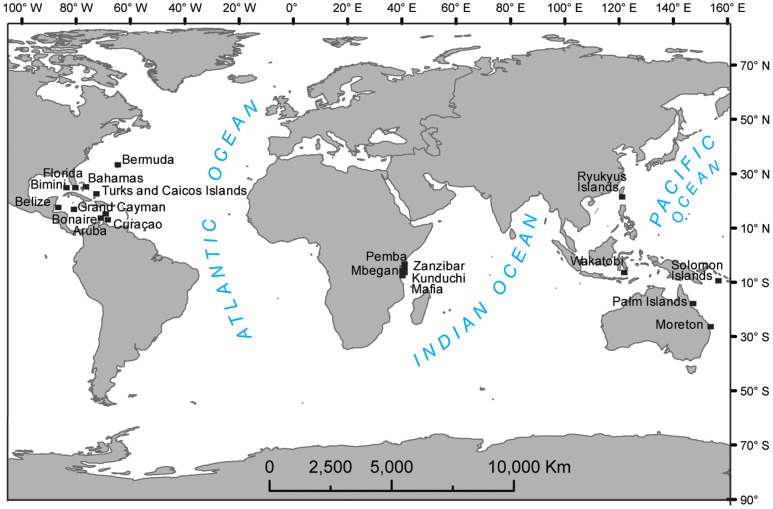
Map showing the study locations for which data were included in the meta-analysis.

There were several selection criteria for inclusion of published articles, but we did not discriminate among fish sampling methods: 1) A particular study must have sampled in more than one habitat: at least in one juvenile habitat (e.g. seagrass beds or mangroves) and including the adult reef habitat. Studies that had sampled artificial seagrass, mangroves or coral reefs were excluded; 2) Studies must have provided mean density data of juvenile fishes, standard deviation (SD) and number of observations (N). In the case where only the standard error of the mean (SE) was provided we calculated SD as the product of SE and square root of the number of observations (N); 3) Studies must have provided length or size range of the juvenile fish sampled; and 4) Each species included in the study must be associated during its juvenile phase with a vegetated habitat other than the adult coral reef habitat, in accordance with the definition of ‘nursery species’ as proposed by others [2], [3]. We based our definition of nursery species on the community-level work of two broad studies [9], [40] for the Caribbean and Indo-Pacific, respectively. However, we included the species groups ‘seagrass generalists’ and ‘reef generalists’ from Dorenbosch et al. [9] because members of these groups have been shown elsewhere to also rely significantly on mangrove and seagrass habitats as juveniles [7], [10], [41]. For our own data (see S1 Table), the size range used to characterize juveniles for the selected species was 0–10 cm TL with exception of the Dorenbosch et al. [9] data where we used a size range based on 0 cm to 1/3^rd^ of the maximum species TL (which has been shown to coincide with the juvenile life phase in [42]). For each study we extracted a single mean density (and associated standard deviation and N) per nursery fish species (juveniles only) per habitat. If the study was done at several study ‘locations’ (e.g. islands) we separated the data per study location (Fig. 1). If studies reported densities across several seasons or various study sites within a bay or estuary, the data were averaged to provide a single mean per species per habitat for each study location. Density measurements were standardized across studies to 100 m^2^ prior to analysis. As most studies did not report mangrove, seagrass, or coral reef cover we could not account for this factor. By far the majority of studies appear to have been conducted in relatively healthy ecosystems.

### Meta-analysis

**Figure 2:**
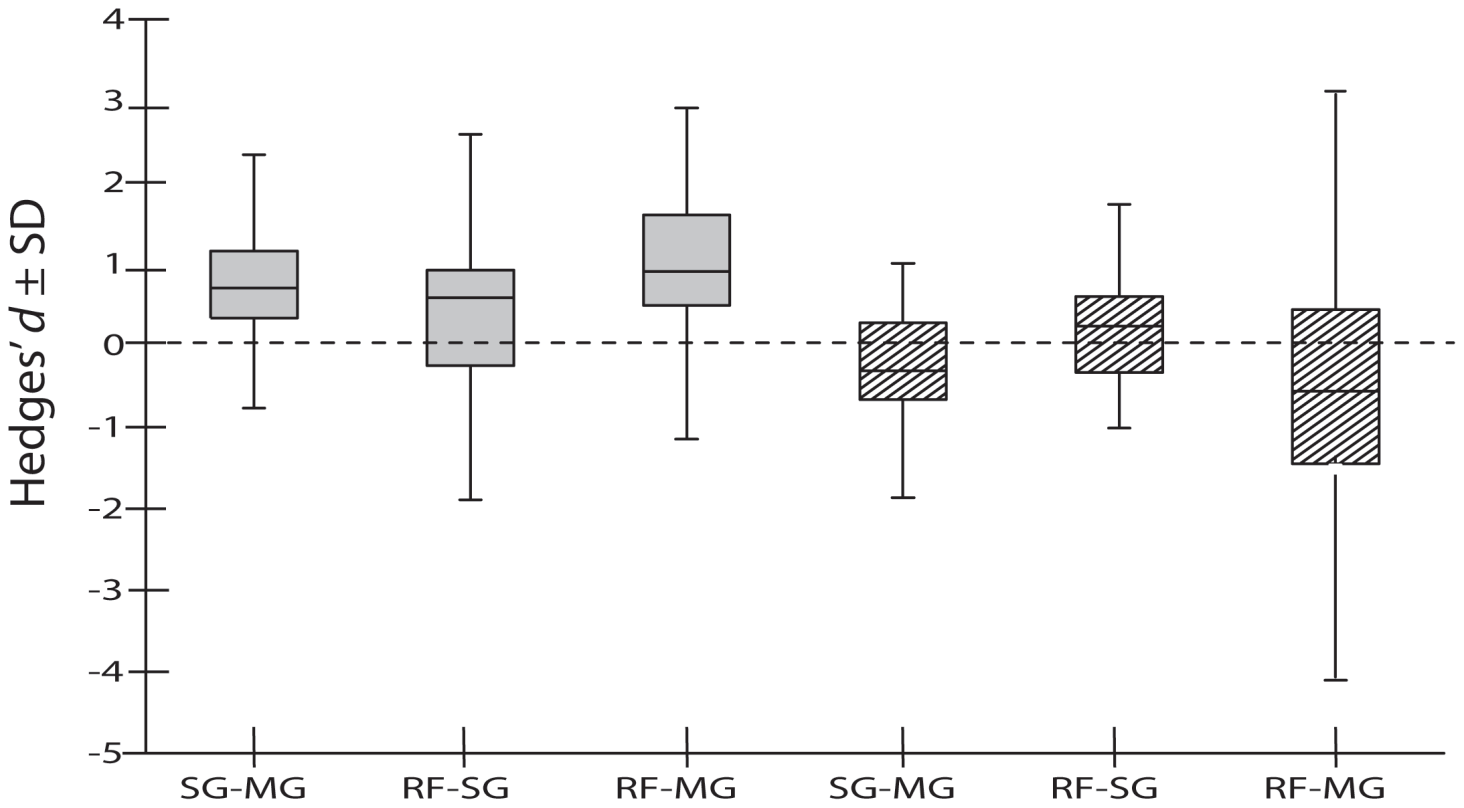
Boxplots showing the distribution of individual effect sizes (Hedges' *d* values) for mean densities of juvenile nursery species in different habitats across locations and fish species. Gray filled boxes indicate data for the Caribbean region, while striped boxes represent the Indo-Pacific region. The boxes show the median effect size (middle line in the box) and the lower and upper quartiles, while the ends of the whiskers represent standard deviation (SD) representing the variability across species and study locations. MG  =  mangroves; SG  =  seagrass beds and RF  =  coral reef. The first mentioned habitat of the pair represents the control habitat for the respective comparison; e.g. SG–MG shows the effect size for fish densities in mangroves (positive  =  higher) compared to seagrass beds. For average Hedges' *d* values, their significance, and associated sensitivity analyses see Table 1.

We used MetaWin version 2.1 [43] for our meta-analysis. Hedges' *d* [44] was used as the metric to measure effect sizes. Hedges' *d* requires a mean, standard deviation (SD) and number of observations (N) for each value to be compared between habitats. Hedges' *d* describes the difference between experimental and control group in terms of SD units. A positive *d* indicates that the experimental group has a larger value than the control group, while a negative value indicates the opposite. For all comparison made (from Fig. 2 onwards), the first mentioned habitat of the pair represents the control habitat for the respective comparison.

Hedges' *d* was calculated for each species at each study location (Supporting Dataset S1). Consecutively, we calculated the cumulative (mean) effect size (*d*_+_) for comparisons across species and study locations (i.e. samples) for each of the three habitat comparisons, using a random-effects model. The cumulative effect size of a sample is a weighted average (weighted by the reciprocal of its sampling variance) of individual effect sizes to reduce bias due to studies with few vs. large sample sizes [43]. Data points (for both species and location) with fewer replicates (N≤2) were excluded in the final analysis.

Confidence intervals around the average effect sizes were generated using bootstrapping methods (5000 iterations). We used biased-corrected confidence intervals to reduce bias due to small sample sizes. If the confidence intervals do not overlap zero, then the effect size is considered significant.

Total heterogeneity (*Q_T_*) of a sample was calculated to determine whether the variance among individual effect sizes calculated for a sample was greater than expected due to sampling error [43]. *Q_T_* is a weighted sum of squares and is comparable to the total sum of squares in an Analysis of Variance (ANOVA). Significance of *Q_T_* was tested against a χ^2^-distribution. A significant *Q_T_* would indicate that other explanatory variables should be investigated as there may be some underlying structure to the data. As the *Q_T_* for all, but one, comparisons was significant (see Table 1) we subsequently ran a categorical random-effects model, which is analogous to a mixed-effects model in ANOVA, using species as a category. In this model, total heterogeneity *Q_T_* is partitioned into *Q_M_* (variation explained by the model) and *Q_E_* (residual error variance). The significance of *Q_M_* was calculated using resampling techniques (based on permutations). Due to the often small sample sizes of meta-analysis, statistics generated through randomization techniques are considered more conservative than parametric methods, because there is no underlying assumption about the distribution of the data [43]. To test the effect of tidal amplitude and salinity on fish habitat use across regions we used a continuous random-effects model with tidal height and water salinity as explanatory variables. These analyses were done using individual data points (i.e. not averaged across species or locations). If tidal range and salinity were not reported in a study, we used sea surface salinity measurements from ocean color (www.oceancolour.gsfc.nasa.gov) and tidal range measurements from tide-forecast (www.tide-forecast.com).

**Table 1:** Summary statistics of a random-effects model for overall comparison of juvenile (1–10 cm, TL) fish densities in seagrass beds (SG), mangroves (MG), and coral reefs (RF).

Region	Comparison	*d*_+_	CI	Bias CI	*Q_T_* (*df*)	p-values	Publication bias test	Fail-safe numbers
**Caribbean**	SG–MG	0.6	0.5 to 0.7*	0.4 to 0.7*	122 (118)	<0.001	Rs (−0.045), *p* (0.631)	R (4551.8)
	RF–SG	0.5	0.3 to 0.6*	0.3 to 0.7*	141.8 (91)	<0.001	Rs (0.068), *p* (0.523)	R (1764.8)
	RF–MG	0.9	0.7 to 1.1*	0.6 to 1.1*	123.0 (107)	0.138	Rs (−0.109), *p* (0.260)	R (6996.1)
**Indo-Pacific**	SG–MG	−0.4	−0.6 to −0.3*	−0.7 to −0.2*	106.9 (57)	<0.001	Rs (0.080), *p* (0.523)	R (2625.1)
	RF–SG	0.1	−0.1 to 0.3	−0.1 to 0.3	84.7 (57)	<0.001	Rs (−0.429), *p* (0.001)	R (539.4)
	RF–MG	−0.3	−0.5 to −0.1*	−0.5 to 0.0*	58.0 (39)	0.026	Rs (−0.065), *p* (0.688)	R (289.3)

Values indicate weighted mean effect size (*d*_+_), 95% confidence intervals (CI), bias-corrected 95% confidence intervals (Bias CI), total heterogeneity (*Q_T_*), and degrees of freedom (*df*). The publication bias test is based on Spearman rank-order correlation (Rs); R =  Rosenthal's methods for calculating fail-safe numbers. Significant p-values indicate significant heterogeneity within a mean effect size; * indicates that the mean effect size is significantly different from zero.

Similarity in habitat use among fish species was analyzed using CLUSTER analysis in PRIMER [45]. Analyses were applied to Euclidean similarity matrices calculated using non-transformed Hedges' *d* values of all three habitat comparisons. CLUSTER analysis was based on group averages.

### Sensitivity analyses

We tested the robustness of the data by calculating Rosenthal's fail-safe numbers for each analysis; this reflects the number of non-significant studies that would need to be added to the analysis to change the outcome from significance to non-significance. A potential caveat of meta-analysis is that of publication bias, caused by selective publication of data (e.g. only with significant outcomes). A rank correlation test (Spearman rank-order) was used to test for publication bias.

## Results

A total of 91 relevant articles were identified from Web of Knowledge and Science Direct, but only 14 articles (with some reporting data from multiple locations) met our criteria for inclusion (S1 Fig.). In total, more study locations were from the relatively small Caribbean region (total: 13) compared to the much larger Indo-Pacific region (total: 10) (Fig. 1, S1 Table). For the latter region, studies were restricted to eastern Africa, Australia, Indonesia, Solomon Islands, and Japan. The sampling method for all included studies was underwater visual census. Tests for publication bias showed no significant effects, except for reef-seagrass in the Indo-Pacific (Table 1). Rosenthal's fail-safe numbers ranged between 289 and 6996 (Table 1), suggesting the results are robust (expected to remain the same if fewer than the above number of non-significant studies are added for the respective comparisons).

In the Caribbean, juvenile densities of nursery species were significantly higher in mangroves than in seagrass beds, while both mangroves and seagrass beds harbored significantly higher juvenile densities of nursery fish compared to coral reefs (Table 1, Fig. 2). By contrast, densities in the Indo-Pacific region were significantly higher in seagrass beds and reefs compared to mangrove habitats. Hence, the overall ranking for most important juvenile habitat was (based on observed densities): mangrove> seagrass> reef in the Caribbean, as opposed to seagrass/reef> mangrove in the Indo-Pacific.

**Figure 3:**
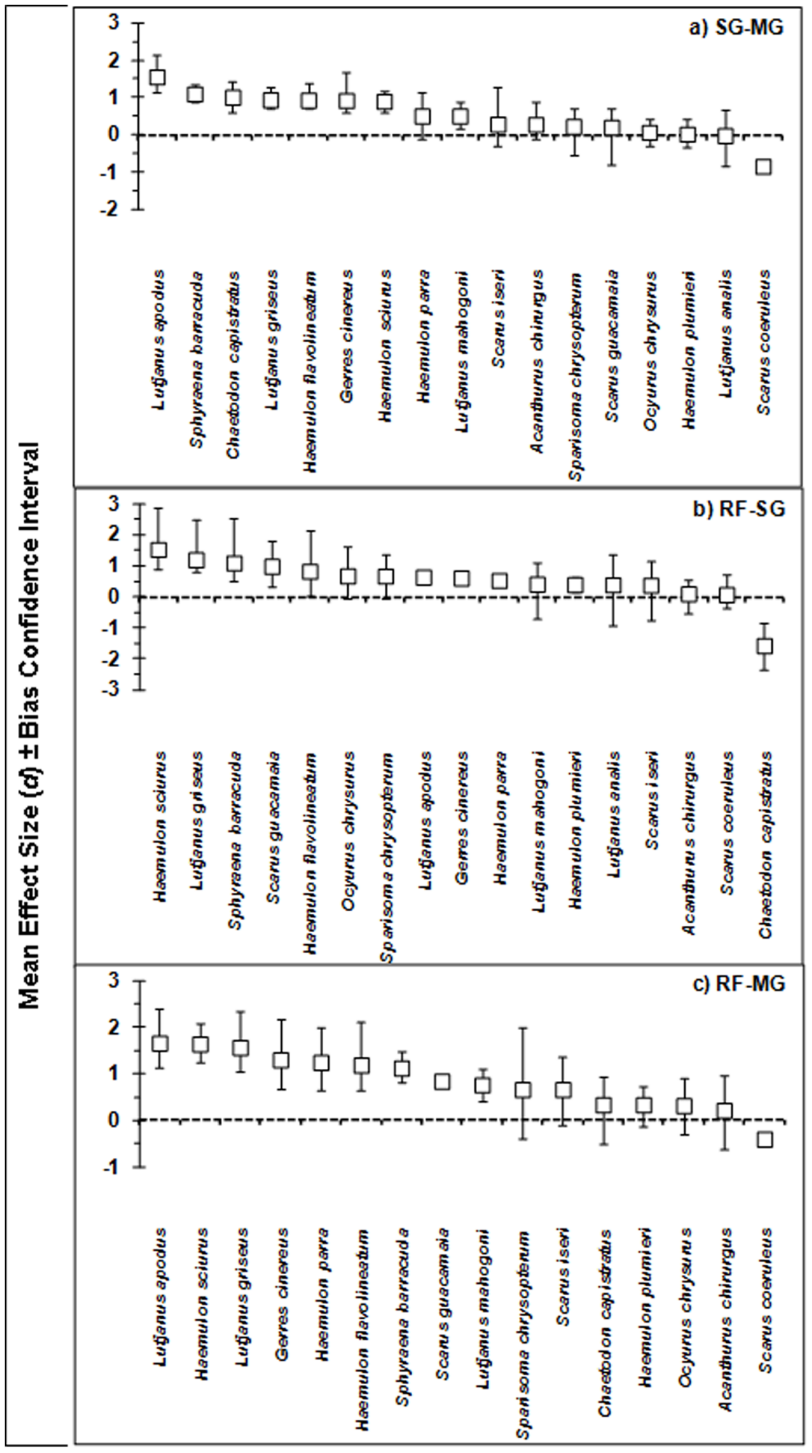
Rank-order of species-specific habitat utilization patterns based on the weighted mean effect size (*d*+) ± bias-corrected 95% confidence interval (based on variability across study locations) for the Caribbean region for a) seagrass (SG) – mangrove (MG) comparison, b) coral reef (RF) – seagrass comparison, and c) coral reef – mangrove comparison. If confidence intervals do not cross the vertical line at *d* = 0, the effect size is significant.

As *Q_T_* was significant for all but one habitat comparison (Table 1) we performed a categorical random-effects analysis with species as categories. Significant differences in habitat utilization were clearly present among species within regions (significant p-values of *Q_M_* ranged between <0.001 and 0.007; S2 Table). In the Caribbean, mangrove habitats were preferentially utilized over seagrass beds for 8 out of 17 species (Fig. 3a). A similar pattern was observed for the reef–mangrove comparison: 9 out of 16 species showed significantly higher densities in mangroves than reef (Fig. 3c), while juvenile *Scarus coeruleus* were more abundant on reef and seagrass beds than in mangroves. For the reef–seagrass comparison there was a significantly higher density in seagrass beds compared to coral reef for 8 out of 17 species, while juvenile *Chaetodon capistratus* were more abundant on the reef than in seagrass beds (Fig. 3b).

**Figure 4:**
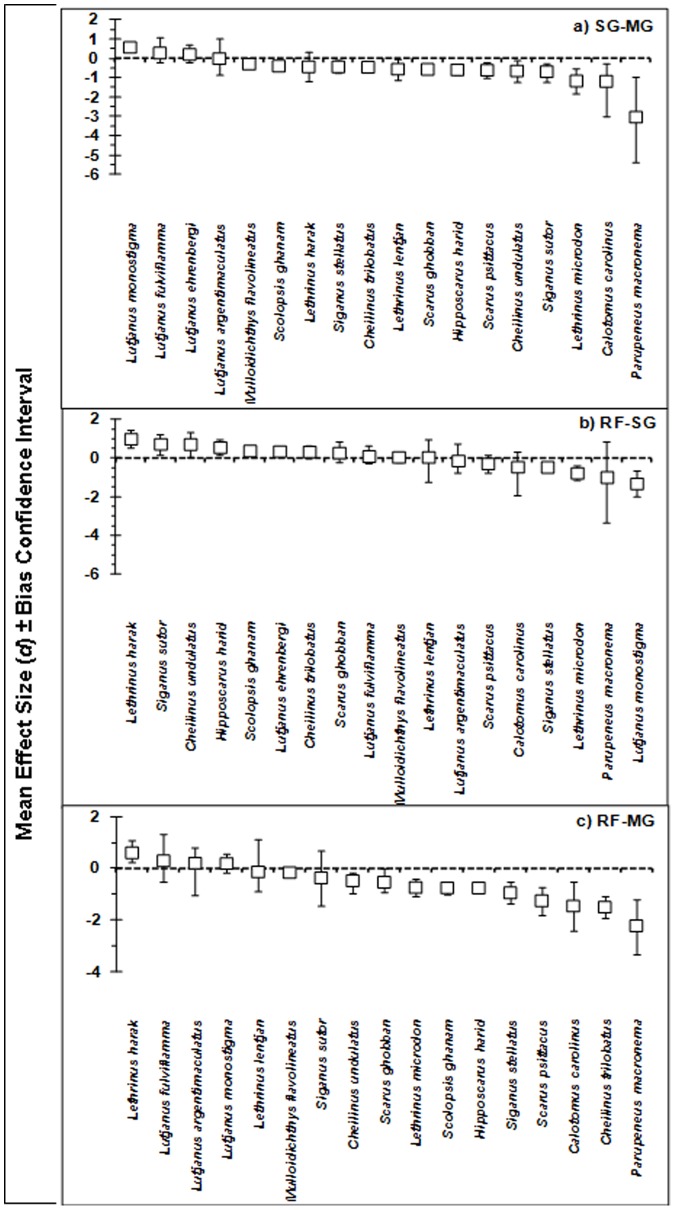
Rank-order of species-specific habitat utilization patterns based on the weighted mean effect size (*d*+) ± bias-corrected 95% confidence interval (based on variability across study locations) for the Indo-Pacific region for a) seagrass (SG) – mangrove (MG) comparison, b) coral reef (RF) – seagrass comparison, and c) coral reef – mangrove comparison. If confidence intervals do not cross the vertical line at *d* = 0, the effect size is significant.

For the Indo-Pacific region, juvenile density of most nursery fish species was lower in mangroves compared to seagrass beds or coral reefs. For instance, 13 out of 18 species had a significantly higher density in seagrass beds compared to mangroves (Fig. 4a) while densities of four other species in seagrass beds were not significantly different from those in mangroves and only one species showed highest densities in mangroves. For the reef–mangrove comparison, 10 species had a significantly higher density on reefs, while densities of six other species did not differ between habitats, and density of only one species was higher in mangroves (Fig. 4c). For the reef–seagrass comparison, seven species had higher densities in seagrass beds vs. three species on reefs (Fig. 4b).

**Figure 5:**
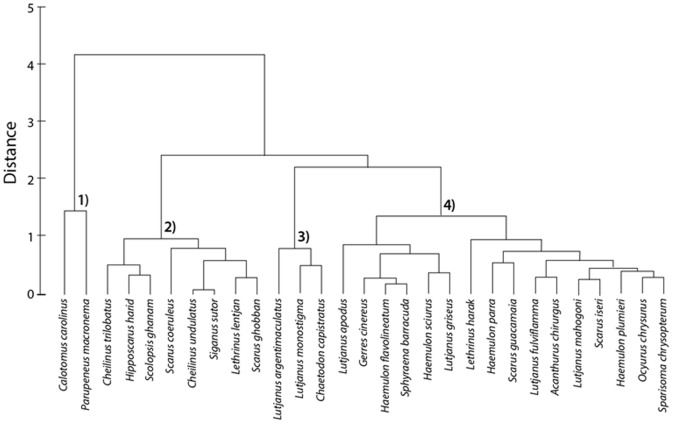
Cluster analysis plot based on Euclidean distances using Hedges' *d* values for all three habitat comparisons per fish species. Species from the Caribbean and Indo-Pacific are combined. Species for which one or more habitat comparisons were absent were omitted as no distance measure could be calculated.

Cluster analysis of effect sizes across habitats separated the species into 4 clusters (Fig. 5): species that were more abundant on reefs than mangrove or seagrass (group 1); species that were abundant on reefs or seagrass, but not in mangroves (group 2); species that were abundant on reefs or mangroves, but not in seagrass (group 3); and species that were abundant in mangroves, but not in seagrass or on reefs (group 4). Most Indo-Pacific species belonged to group 2 (not abundant in mangroves), whereas most Caribbean species belonged to group 4 (abundant in mangroves). The few Indo-Pacific species that occurred in high densities in mangroves did not co-occur in high densities in seagrass beds as was the case for some Caribbean species. Overall, species that were found in mangroves in high densities were dominated by the families Haemulidae (grunts), Lutjanidae (snappers), and Scaridae (parrotfishes).

**Figure 6:**
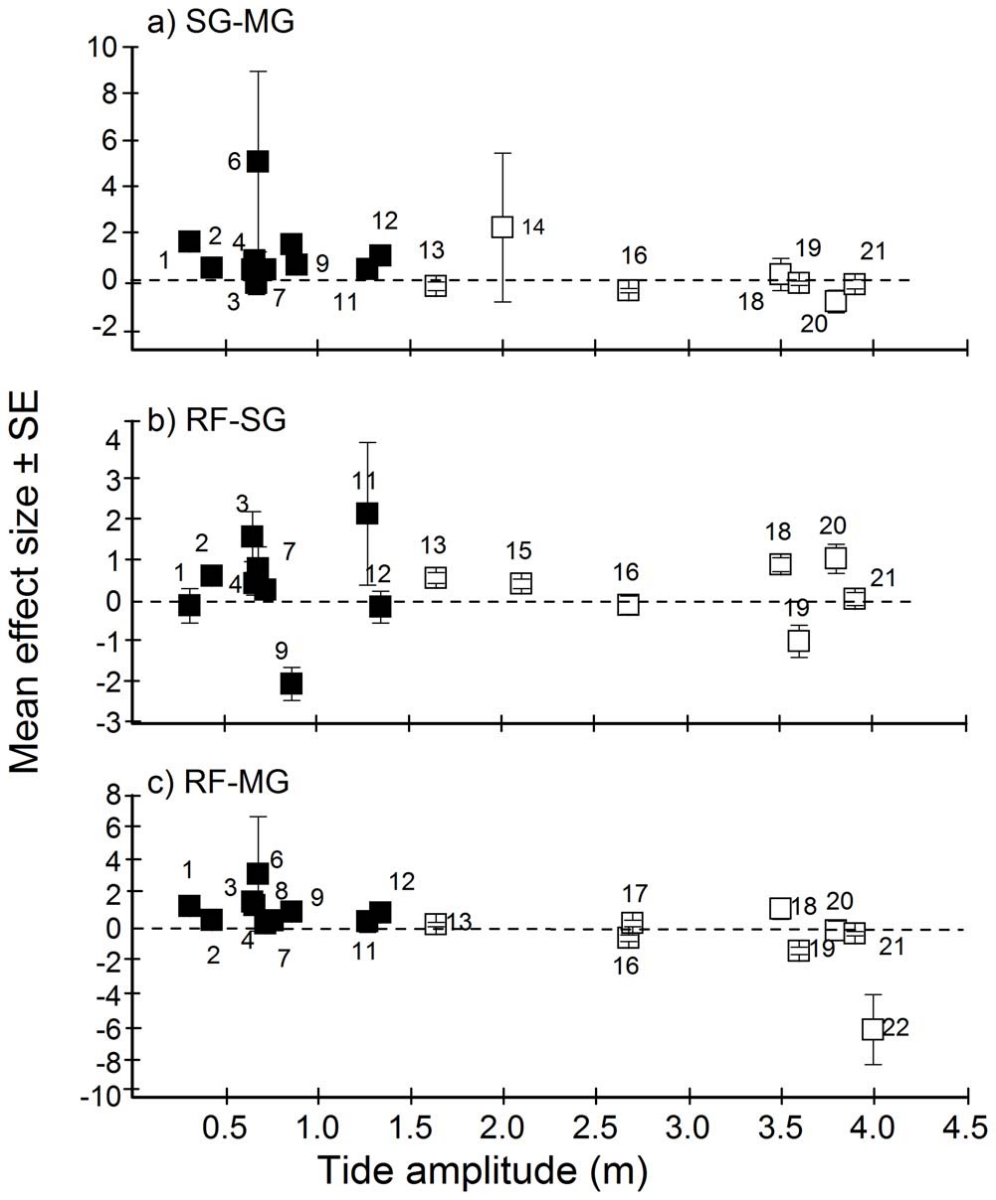
Mean effect size ± SE (based on variability across species) for densities of juvenile nursery species in different habitats as a function of tidal amplitude for the Caribbean (filled squares) and Indo-Pacific (open squares) regions, for a) seagrass (SG) – mangrove (MG) comparison, b) coral reef (RF) – seagrass comparison, and c) coral reef – mangrove comparison. Numbers in the graphs indicate: Belize (1), Curaçao (2), Aruba (3), Grand Cayman (4), Florida (5), Turks and Caicos Islands (6), Bimini (7), San Salvador (8), Andros (9), Abaco (10), Bermuda (11), Lee Stocking Island (12), Solomon Islands (13), Wakatobi (14), Ryukyu Islands (15), Moreton (16), Palm Islands (17), Kunduchi (18), Mafia (19), Mbegani (20) Zanzibar (21) and Pemba (22).

Densities of juvenile nursery species showed a significant correlation with tidal regime. With increasing tidal amplitude, densities in seagrass beds (df  = 148; slope  = −0.1852; p<0.001) and mangroves (df  = 182; slope  = −0.6360; p<0.001) decreased compared to coral reefs, while in mangroves (df  = 169; slope  = −0.2740; p<0.001) they decreased compared to seagrass beds (Fig. 6). Because all small-tidal data points originated from the Caribbean and all large-tidal data are from the Indo-Pacific, we also performed a continuous random-effects analysis within regions. For the Caribbean no significant effect of tide was found for any of the habitat comparisons (all p>0.303), likely due to the small range (0.3–1.3 m) in tides across islands tested. For the Indo-Pacific, however, where a large range (1.6–4.0 m) in tidal amplitudes across locations was tested, a significant effect of tide was found for the reef–seagrass comparison (df  = 56; slope  = −0.2733; p = 0.050) and the reef–mangrove comparison (df  = 74; slope  = −0.8450; p<0.001), but not for the seagrass–mangrove comparison (df  = 50; slope  = 0.0321; p = 0.730). Finally, for the seagrass–mangrove comparison juvenile fish densities showed a significant correlation with salinity, with higher densities in mangroves with increased salinity (df  = 169; slope  = 0.1952; p = 0.005).

## Discussion

The importance of Indo-Pacific mangroves as nursery habitats for juvenile coral reef fishes has been debated for decades (see [4], [46] for reviews). Yet, emerging evidence shows that some Indo-Pacific mangroves may function as nurseries for some species as much as their Caribbean counterparts do [47], [48]. The inherent nursery value of Indo-Pacific seagrass has been accepted more readily, but is still often overlooked in marine conservation [48]–[50]. The present study used a meta-analysis approach to examine circumtropical habitat use patterns by juveniles of nursery reef fish species. We found relatively few published studies that compared the abundance of juvenile nursery fish in two or more juvenile habitats, and which also examined the adult habitat. Many studies are still based on observations from single juvenile habitats (see for example [51]–[53]), or do not separate juvenile from adult densities if multiple habitats are studied, or exclude adult habitats from surveys. These omissions have made it difficult to quantify the relative usage of different habitats by juvenile fish nursery species.

Our meta-analysis shows that mangroves harbor the highest juvenile densities for most nursery species in the Caribbean, whereas seagrass beds appear to be the primary juvenile fish habitat in the Indo-Pacific region. It appears that the small tidal amplitude in the Caribbean as opposed to the large amplitude in the Indo-Pacific region plays an important role in driving this pattern of mangrove versus seagrass use, with juvenile fish being provided with longer access to mangroves in micro-tidal areas [27], [54]. In the Caribbean, narrow fringing mangroves typically provide ideal shelter, but less feeding opportunities for juvenile fish compared to adjacent seagrass beds [55]. Many of the species observed in mangroves during daytime move to seagrass beds at night to feed [56], [57]. Our density data are based on daytime observations and hence the higher densities in mangroves compared to seagrass beds in the Caribbean. In the Indo-Pacific, mangroves are often too shallow at low tide limiting the ability of fish to remain in mangroves throughout tidal cycles. For some species more food resources may be present in mangroves, although predation risk and growth were not found to be elevated in fish that use this habitat preferentially over seagrass beds [11], [58]. Moreover, movement between habitats is risky [59] and may outweigh any benefits gained from visiting mangroves. It is therefore likely that fewer reef species have adapted to mangrove utilization in macro-tidal areas where mangrove and seagrass habitats co-occur. This is supported by our meta-analysis which showed that, compared to the Caribbean, a much lower number of nursery species (just three) preferentially used mangroves over other habitats in the Indo-Pacific region, even though the latter is a much more speciose biogeographic region than the Caribbean. The large tidal fluctuations may put physiological and energetic limitations on species entering this habitat and this may have contributed to a reduced dependence on this habitat in the Indo-Pacific [27], [60]. Our findings suggest that tidal amplitude is globally a strong driver of the number of reef species and individuals that make significant use of mangrove habitats in seascapes that consist of mangrove-seagrass mosaics.

Apart from tidal amplitude, also salinity appears to exert a significant effect on fish densities, with densities increasing in mangroves relative to seagrass beds in areas with higher salinities. Mangroves are often located in estuaries and there is emerging evidence that estuarine mangroves are frequented more by estuarine fishes than juvenile coral reef fishes, whereas the opposite is true for marine mangroves [6], [61], [62]. Non-estuarine mangroves may, therefore, be more attractive or beneficial to larvae of marine than estuarine species. The structural complexity of mangrove roots is also often favored by juvenile fish over seagrasses [63], which may explain the increased usage of mangroves over seagrass beds at higher salinities.

Mangrove inundation patterns and salinity gradients in bays and estuaries may change due rising sea levels and changes in precipitation as a result of climate change. It will be difficult to predict how such modifications to physic-chemical factors due to climate change may alter mangrove habitat use by fishes, and this will be highly dependent on local topographical, environmental and anthropogenic influences. Sea level rise may increase water depth in some mangroves and thus increase their accessibility. However, this may not be the case where mangroves migrate to higher elevations, or in cases where their sediment accretion rates keep up with rising sea levels [64]. Failure of such responses may lead to a rearrangement of vegetation types or loss of mangroves, especially at the seaward fringe, due increased erosion and intolerance to extended exposure to salt water. Mangroves located in estuaries might be able to cope better with increasing sea levels than non-estuarine mangroves due to sufficient allochthonous sediment input from rivers [65]. On the other hand, in regions where rainfall is predicted to increase, estuarine mangroves may experience an overall decrease in salinity levels [33] making them less suitable for juveniles of marine species.

Overall, there seems to be a tendency of higher mangrove reliance or utilization by various members of the families Haemulidae, Lutjanidae, and Sphyraenidae during their juvenile stages, independent of biogeographic locality. This included species like *Sphyraena barracuda*, *Scarus guacamaia*, *Lutjanus apodus*, *Lutjanus griseus, Gerres cinereus, Haemulon flavolineatum*, *Haemulon sciurus,* and *Haemulon parra* for the Caribbean region and *Lutjanus argentimaculatus*, *Lutjanus fulviflamma* and *Lutjanus monostigma* for the Indo-Pacific region. Potentially this could be related to specific life-history traits that are characteristic of some species within these fish families. For instance, two species of Indo-Pacific haemulid juveniles (*Plectorhinchus albovittatus* and *P. aibbosus*) have been observed in mangroves, whereas most other members of the *Plectorhinchus* genus occur in seagrass or on reef as juveniles [66]. Also lutjanids and Caribbean haemulids are represented by nursery as well as non-nursery species.

The fact that mangrove and/or seagrass habitats harbor high densities of juvenile fish does not rule out the possibility that other shallow water habitats are also used as juvenile habitats. The effect sizes showed relatively large confidence intervals for some species, indicating that the magnitude of seagrass and mangrove habitat use differed for various species across locations within the two regions. This is likely due to complex local-scale interactions between species abundances/size classes, and seascape structure and connectivity. Such interactions make it difficult to single out seagrass beds and/or mangroves alone as primary juvenile habitats, especially in areas where mangroves and seagrass beds are interconnected with each other and/or with coral reefs through diurnal, tidal, seasonal, or ontogenetic movements [36]. Juvenile nursery fish species may use multiple habitats depending on their availability and accessibility [4], [67]. The usage of multiple habitats suggests flexibility, which can be linked to ontogenetic habitat shifts (e.g. for feeding and shelter) and different habitat functions at different spatial and temporal scales [7], [14]. This strategy of shifting among habitats throughout ontogeny is thought to maximize a trade-off between growth and survival [68]. However, specific habitat preference and use can change with even small increases in body size. For example, juveniles of *Haemulon* spp. settle in rubble zones, then move to seagrass beds followed by mangroves and rocky substratum, before they move to the adult coral reef habitat [58], [69]. Finally, it is not only the density of fish that ultimately determines the contribution to the adult habitat, but also the total surface area of the juvenile habitat in question [1], aside from other drivers such as growth, survival and movement [2].

The results of meta-analyses can potentially be influenced by a number of factors. These include differences in sampling methods among studies, publication bias and both spatial and temporal variability in study context (e.g. habitat proximity/connectivity) and timing (e.g. sampling season). For example, studies conducted during recruitment periods may provide different results and thus affect the outcome. The studies that met our criteria were all based on visual surveys, performed at multiple locations (averaged for the meta-analysis), and we tested for publication bias. Therefore, it seems unlikely that these factors had a large effect on the results. Variation in seascape structure among studies could have had an effect on fish habitat use, explaining some of the variability among studies. The incorporation of seascape variables, along with spatial information on fish densities and movement, into analyses of juvenile habitat use will provide an important step towards better predicting the value of nursery habitats [36].

### Conclusions

Our global analysis of potential nursery habitat use suggests that mangroves are the preferred juvenile habitat for many nursery species in the Caribbean, whereas seagrass beds seem to fulfill this role in the Indo-Pacific. Tidal regime appears to act as an important driver of these patterns of fish distributions and degree of habitat connectivity, with the relative usage of mangroves decreasing with increasing tidal amplitude. Salinity may play an additional important role in mangrove habitats, restricting their use by juvenile reef fish in more estuarine conditions. To prioritize the management and conservation of these key juvenile habitats it is critical to consider the overarching role that tidal regime can play on patterns of fish habitat use, and how this may be affected by sea-level rise due to climate change.

## Supporting Information

S1 Fig **Flow of information through the different phases of the meta-analysis.**
(TIF)

S1 Table List of studies from the literature and our own data sources used in the analyses.
(DOCX)

S2 Table Random-effects categorical model summary statistics to test for the effect of species identity within habitat comparisons.
(DOCX)

S1 Dataset **Hedges'** ***d*** **values and their variance calculated with MetaWin for the comparison of fish densities among mangroves, seagrass beds and coral reefs.**
(XLSX)

S1 Checklist (DOC)

## References

[1] Dahlgren CP, Kellison GT, Adams AJ, Gillanders BM, Kendall MS, et al (2006) Marine nurseries and effective juvenile habitats: concepts and applications. Marine Ecology Progress Series 312:291–295.

[2] Beck JD, Heck LK, Able KW, Childers DL, Eggleston DB, et al (2001) The identification, conservation, and management of estuarine and marine nurseries for fish and invertebrates. Bioscience 51:633–641.

[3] Adams AJ, Dahlgren CP, Kellison GT, Kendall MS, Layman CA, et al (2006) Nursery function of tropical back-reef systems. Marine Ecology Progress Series 318:287–301.

[4] Nagelkerken I (2009) Evaluation of nursery function of mangroves and seagrass beds for tropical decapods and reef fishes: patterns and underlying mechanisms. In: Nagelkerken I, editor. Ecological connectivity among tropical coastal ecosystems. Dordrecht, The Netherlands: Springer Science and Business Media. pp.357–399

[5] Leis JM, McCormick MI (2002) The Biology, behavior and ecology of the pelagic larval stage of coral reef fishes. In: Sale P, Feditor. Coral Reef Fishes. San Diego: Academic Press. pp.171–199.

[6] Nagelkerken I (2007) Are non-estuarine mangroves connected to coral reefs through fish migration? Bulletin of Marine Science 80:595–607.

[7] Kimirei IA, Nagelkerken I, Griffioen B, Wagner C, Mgaya YD (2011) Ontogenetic habitat use by mangrove/seagrass-associated coral reef fishes shows flexibility in time and space. Estuarine, Coastal and Shelf Science 92:47–58.

[8] Minello TJ, Able KW, Weinstein MP, Hays CG (2003) Salt marshes as nurseries for nekton: testing hypotheses on density, growth and survival through meta-analysis. Marine Ecology Progress Series 246:39–59.

[9] Dorenbosch M, Nagelkerken I, Grol MGG, Christianen MJA, van der Velde G (2005) Indo-Pacific seagrass beds and mangroves contribute to fish density coral and diversity on adjacent reefs. Marine Ecology Progress Series 302:63–76.

[10] Heck KL, Hays G, Orth RJ (2003) Critical evaluation of the nursery role hypothesis for seagrass meadows. Marine Ecology Progress Series 253:123–136.

[11] Kimirei IA, Nagelkerken I, Mgaya YD, Huijbers CM (2013) The Mangrove Nursery Paradigm Revisited: Otolith Stable Isotopes Support Nursery-to-Reef Movements by Indo-Pacific Fishes. PloS one 8:e66320. 23776658 10.1371/journal.pone.0066320 PMC3680401

[12] Nyunja J, Ntiba M, Onyari J, Mavuti K, Soetaert K, et al (2009) Carbon sources supporting a diverse fish community in a tropical coastal ecosystem (Gazi Bay, Kenya). Estuarine, Coastal and Shelf Science 83:333–341.

[13] Heithaus ER, Heithaus PA, Heithaus MR, Burkholder D, Layman CA (2011) Trophic dynamics in a relatively pristine subtropical fringing mangrove community. Marine Ecology Progress Series 428:49–61.

[14] Igulu M, Nagelkerken I, van der Velde G, Mgaya Y (2013) Mangrove fish production is largely fuelled by external food sources: A stable isotope analysis of fishes at the individual, species, and community levels from across the globe. Ecosystems 16:1336–1352.

[15] Nagelkerken I, Kleijnen S, Klop T, van den Brand RACJ, Cocheret de la Morinière E, et al (2001) Dependence of Caribbean reef fishes on mangroves and seagrass beds as nursery habitat: a comparison of fish faunas between bays with and without mangroves/seagrass beds. Marine Ecology Progress Series 214:225–235.

[16] Mumby PJ, Edwards AJ, Arias-Gonzalez JE, Lindeman KC, Blackwell PG, et al (2004) Mangroves enhance the biomass of coral reef fish communities in the Caribbean. Nature 427:533–536. 14765193 10.1038/nature02286

[17] Mumby PJ, Hastings A (2008) The impact of ecosystem connectivity on coral reef resilience. Journal of Applied Ecology 45:854–862.

[18] Olds AD, Pitt KA, Maxwell PS, Connolly RM (2012) Synergistic effects of reserves and connectivity on ecological resilience. Journal of Applied Ecology 49:1195–1203.

[19] Sheridan P (1997) Benthos of adjacent mangrove, seagrass and non-vegetated habitats in Rookery Bay, Florida, USA. Estuarine, Coastal and Shelf Science 44:455–469.

[20] Lee SY (1995) Mangrove outwelling - A review. Hydrobiologia 295:203–212.

[21] Laegdsgaard P, Johnson C (2001) Why do juvenile fish utilise mangrove habitats? Journal of Experimental Marine Biology and Ecology 257:229–253. 11245878 10.1016/s0022-0981(00)00331-2

[22] Unsworth RKF, Bell JJ, Smith DJ (2007) Tidal fish connectivity of reef and sea grass habitats in the Indo-Pacific. Journal of the Marine Biological Association of the United Kingdom 87:1287–1296.

[23] Nagelkerken I, Blaber SJM, Bouillon S, Green P, Haywood M, et al (2008) The habitat function of mangroves for terrestrial and marine fauna: A review. Aquatic Botany 89:155–185.

[24] Olds AD, Connolly RM, Pitt KA, Maxwell PS (2012) Habitat connectivity improves reserve performance. Conservation Letters 5:56–63.

[25] Dorenbosch M, Verweij MC, Nagelkerken I, Jiddawi N, van der Velde G (2004) Homing and daytime tidal movements of juvenile snappers (Lutjanidae) between shallow-water nursery habitats in Zanzibar, western Indian Ocean. Environmental Biology of Fishes 70:203–209.

[26] Thompson AA, Mapstone BD (2002) Intra- versus inter-annual variation in counts of reef fishes and interpretations of long-term monitoring studies. Marine Ecology Progress Series 232:247–257.

[27] Sheaves M (2005) Nature and consequences of biological connectivity in mangrove systems. Marine Ecology Progress Series 302:293–305.

[28] Dorenbosch M, Verberck WCEP, Nagelkerken I, van der Velde G (2007) Influence of habitat configuration on connectivity between fish assemblages of Caribbean seagrass beds, mangroves and coral reefs. Marine Ecology Progress Series 334:103–116.

[29] Nagelkerken I, Grol MGG, Mumby PJ (2012) Effects of marine reserves versus nursery habitat availability on structure of reef fish communities. Plos One 7:e36906. 22675474 10.1371/journal.pone.0036906 PMC3366965

[30] Barletta M, Barletta-Bergan A, Saint-Paul U, Hubold G (2005) The role of salinity in structuring the fish assemblages in a tropical estuary. Journal of Fish Biology 66:45–72.

[31] Neves LM, Teixeira TP, Araujo FG (2011) Structure and dynamics of distinct fish assemblages in three reaches (upper, middle and lower) of an open tropical estuary in Brazil. Marine Ecology-an Evolutionary Perspective 32:115–131.

[32] Whitfield AK, Taylor RH, Fox C, Cyrus DP (2006) Fishes and salinities in the St Lucia estuarine system—a review. Reviews in Fish Biology and Fisheries 16:1–20.

[33] Gillanders BM, Elsdon TS, Halliday IA, Jenkins GP, Robins JB, et al (2011) Potential effects of climate change on Australian estuaries and fish utilising estuaries: a review. Marine & Freshwater Research 62:1115–1131.

[34] Harrison TD, Whitfield AK (2006) Temperature and salinity as primary determinants influencing the biogeography of fishes in South African estuaries. Estuarine Coastal and Shelf Science 66:335–345.

[35] Sheridan P, Hays C (2003) Are mangroves nursery habitat for transient fishes and decapods? Wetlands 23:449–458.

[36] Nagelkerken I, Sheaves M, Baker R, Connolly RM (2015) The seascape nursery: a novel spatial approach to identify and manage nurseries for coastal marine fauna. Fish and Fisheries. DOI: 10.1111/faf.12057.

[37] Shibuno T, Nakamura Y, Horinouchi M, Sano M (2008) Habitat use patterns of fishes across the mangrove-seagrass-coral reef seascape at Ishigaki Island, southern Japan. Ichthyological Research 55:218–237.

[38] Nakamura Y, Sano M (2004) Overlaps in habitat use of fishes between a seagrass bed and adjacent coral and sand areas at Amitori Bay, Iriomote Island, Japan: Importance of the seagrass bed as juvenile habitat. Fisheries Science 70:788–803.

[39] Sanchirico JN, Mumby PJ (2009) Mapping ecosystem functions to the valuation of ecosystem services: implications of species-habitat associations for coastal land-use decisions. Theoretical Ecology 2:67–77.

[40] Nagelkerken I, Dorenbosch M, Verberk WCEP, Cocheret de la Morinière E, van der Velde G (2000) Importance of shallow-water biotopes of a Caribbean bay for juvenile coral reef fishes: patterns in biotope association, community structure and spatial distribution. Marine Ecology Progress Series 202:175–192.

[41] Nakamura Y, Shibuno T, Lecchini D, Kawamura T, Watanabe Y (2009) Spatial variability in habitat associations of pre- and post-settlement stages of coral reef fishes at Ishigaki Island, Japan. Marine Biology 156:2413–2419.

[42] Nagelkerken I, van der Velde G (2002) Do non-estuarine mangroves harbour higher densities of juvenile fish than adjacent shallow-water and coral reef habitats in Curacao (Netherlands Antilles)? Marine Ecology Progress Series 245:191–204.

[43] Rosenberg MS, Adams DC, Gurevitch J (2000) Metawin: Statistical software for meta-analysis. Version 2.1. Sinauer Associates, Sunderland, Massachusetts.

[44] Hedges LV, Olkin I (1985) Statistical methods for meta-analysis. New York: Academic Press.

[45] Clarke KR, Gorley RN (2006) PRIMERv6: user manual/tutorial. PRIMER-E. Plymouth Marine Laboratory: Plymouth, UK.

[46] Faunce CH, Layman CA (2009) Sources of variation that affect perceived nursery function of mangroves. In: Nagelkerken Ieditor. Ecological Connectivity among Tropical Coastal Ecosystems. Dordrecht: Springer Science + Bussiness Media. pp.401–421.

[47] Nakamura Y, Tsuchiya M (2008) Spatial and temporal patterns of seagrass habitat use by fishes at the Ryukyu Islands, Japan. Estuarine, Coastal and Shelf Science 76:345–356.

[48] Olds AD, Albert S, Maxwell PS, Pitt KA, Connolly RM (2013) Mangrove-reef connectivity promotes the effectiveness of marine reserves across the western Pacific. Global Ecology and Biogeography 22:1040–1049.

[49] Kimirei I, Nagelkerken I, Trommelen M, Blankers P, van Hoytema N, et al (2013) What Drives Ontogenetic Niche Shifts of Fishes in Coral Reef Ecosystems? Ecosystems 16:783–796.

[50] Unsworth RKF, Cullen LC (2010) Recognising the necessity for Indo-Pacific seagrass conservation. Conservation Letters 3:63–73.

[51] Crona BI, Ronnback P (2005) Use of replanted mangroves as nursery grounds by shrimp communities in Gazi Bay, Kenya. Estuarine, Coastal and Shelf Science 65:535–544.

[52] Crona BI, Ronnback P (2007) Community structure and temporal variability of juvenile fish assemblages in natural and replanted mangroves, Sonneratia alba Sm., of Gazi Bay, Kenya. Estuarine, Coastal and Shelf Science 74:44–52.

[53] Mwandya AW, Gullström M, Andersson MH, Öhman MC, Mgaya YD, et al (2010) Spatial and seasonal variations of fish assemblages in mangrove creek systems in Zanzibar (Tanzania). Estuarine, Coastal and Shelf Science 89:277–286.

[54] Krumme U (2009) Diel and tidal movements by fish and decapods linking tropical coastal ecosystems. In: Nagelkerken Ieditor. Ecological connectivity among tropical coastal ecosystems. New York: Springer. pp.271–324.

[55] Verweij MC, Nagelkerken I, Wartenbergh SLJ, Pen IR, van der Velde G (2006) Caribbean mangroves and seagrass beds as daytime feeding habitats for juvenile French grunts, *Haemulon flavolineatum* . Marine Biology 149:1291–1299.

[56] Nagelkerken I, Dorenbosch M, Verberk WCEP, Cocheret de la Morinière E, van der Velde G (2000) Day-night shifts of fishes between shallow-water biotopes of Caribbean bay, with emphasis on the nocturnal feeding of Haemulidae and Lutjanidae. Marine Ecology Progress Series 194:55–64.

[57] Hammerschlag N, Heithaus MR, Serafy JE (2010) Influence of predation risk and food supply on nocturnal fish foraging distributions along a mangrove-seagrass ecotone. Marine Ecology Progress Series 414:223–235.

[58] Grol MGG, Rypel AL, Nagelkerken I (2014) Growth potential and predation risk drive ontogenetic shifts among nursery habitats in a coral reef fish. Marine Ecology Progress Series 502:229–244.

[59] Gilliam JF, Fraser DF (2001) Movement in corridors: Enhancement by predation threat, disturbance, and habitat structure. Ecology 82:258–273.

[60] Jelbart JE, Ross PM, Connolly RM (2007) Fish assemblages in seagrass beds are influenced by the proximity of mangrove forests. Marine Biology 150:993–1002.

[61] Lugendo BR, Nagelkerken I, Kruitwagen G, van der Velde G, Mgaya YD (2007) Relative importance of mangroves as feeding habitats for fishes: A comparison between mangrove habitats with different settings. Bulletin of Marine Science 80:497–512.

[62] Thollot P (1992) Importance of mangroves for Pacific reef fish species, myth or reality? Proceeding of the 7^th^ International Coral Reef Symposium pp. 934–941.

[63] Verweij MC, Nagelkerken I, de Graaff D, Peeters M, Bakker EJ, et al (2006) Structure, food and shade attract juvenile coral reef fish to mangrove and seagrass habitats: a field experiment. Marine Ecology Progress Series 306:257–268.

[64] Field CD (1995) Impact of expected climate-change on mangroves. Hydrobiologia 295:75–81.

[65] Ellison JC, Stoddart DR (1991) Mangrove ecosystem collapse during predicted sea-level rise- Holocene analogs and implications. Journal of Coastal Research 7:151–165.

[66] Olds AD, Connolly MR, Pitt KA, Maxwell PS, Aswani S, et al (2014) Incorporating surrogate species and seascape connectivity to improve marine conservation outcomes. Conservation Biology 28:982–991. 24527964 10.1111/cobi.12242

[67] Verweij MC, Nagelkerken I (2007) Short and long-term movement and site fidelity of juvenile Haemulidae in back-reef habitats of a Caribbean embayment. Hydrobiologia 592:257–270.

[68] Dahlgren CP, Eggleston DB (2000) Ecological processes underlying ontogenetic habitat shifts in a coral reef fish. Ecology 81:2227–2240.

[69] Grol MGG, Nagelkerken I, Rypel AL, Layman CA (2011) Simple ecological trade-offs give rise to emergent cross-ecosystem distributions of a coral reef fish. Oecologia 165:79–88. 21072542 10.1007/s00442-010-1833-8 PMC3015207

[70] Harborne AR, Mumby PJ, Kappel CV, Dahlgren CP, Micheli F, et al (2008) Tropical coastal habitats as surrogates of fish community structure, grazing and fisheries value. Ecological Applications 18:1689–1701. 18839764 10.1890/07-0454.1

[71] Harborne AR, Mumby PJ, Kappel CV, Dahlgren CP, Micheli F, et al (2008) Reserve effects and natural variation in coral reef communities. Journal of Applied Ecology 45:1010–1018.

[72] Huijbers CM, Grol MGG, Nagelkerken I (2008) Shallow patch reefs as alternative habitats for early juveniles of some mangrove/seagrass-associated fish species in Bermuda. Revista de Biologia Tropical 56:161–169.

[73] Sheridan PF (1992) Comparative habitat utilization by estuarine macrofauna within the mangrove ecosytem of Rookery bay, Florida. Bulletin of Marine Science 50:21–39.

[74] Olds AD, Connolly RM, Pitt KA, Maxwell PS (2012) Primacy of seascape connectivity effects in structuring coral reef fish assemblages. Marine Ecology Progress Series 462:191–203.

[75] Unsworth RKF, Garrard SL, De Leon PS, Cullen LC, Smith DJ, et al (2009) Structuring of Indo-Pacific fish assemblages along the mangrove-seagrass continuum. Aquatic Biology 5:85–95.

