# Supplementary figures and images for: Mangrove Habitat Use by Juvenile Reef Fish: Meta-Analysis Reveals that Tidal Regime Matters More than Biogeographic Region
Source: PLoS One. 2014 Dec 31;9(12):e114715. doi: 10.1371/journal.pone.0114715 (PMC4281128; doi:10.1371/journal.pone.0114715)

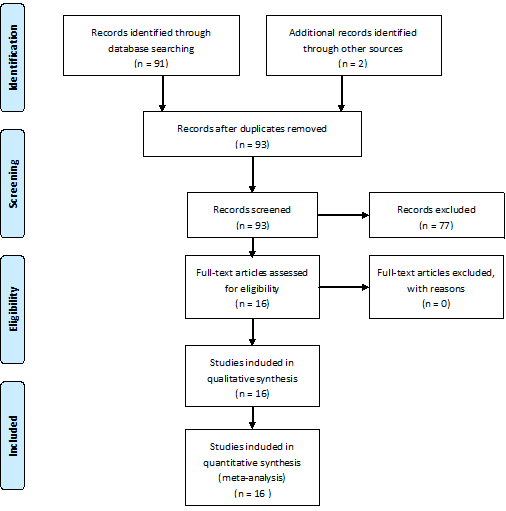

Supplement: S1 Fig — Flow of information through the different phases of the meta-analysis. (TIF) [file pone.0114715.s001.tif]
