# Supplementary material for: Mangrove Habitat Use by Juvenile Reef Fish: Meta-Analysis Reveals that Tidal Regime Matters More than Biogeographic Region
Source: PLoS One. 2014 Dec 31;9(12):e114715. doi: 10.1371/journal.pone.0114715 (PMC4281128; doi:10.1371/journal.pone.0114715)
Supplement: S1 Table — List of studies from the literature and our own data sources used in the analyses. (DOCX) [file pone.0114715.s002.docx]

**Supplementary Material**

Table S1. List of studies from the literature and our own data sources (*) used in the analyses. MG = mangroves, SG = seagrass beds and RF = coral reef.

| **Reference** | **Region / location** | **Habitat sampled** | **Number of nursery species** |
| --- | --- | --- | --- |
|  | **Caribbean** |  |  |
| Dorenbosch et al. [28] | Aruba | MG, SG, RF | 17 |
| Harborne et al. [70] | Abaco (Bahamas) | MG, SG | 17 |
| Harborne et al. [70] | Andros (Bahamas) | MG, SG, RF | 17 |
| Harborne et al. [70] | Bimini (Bahamas) | MG, SG | 17 |
| Nagelkerken unpubl. data* | Bimini (Bahamas) | MG, SG, RF | 17 |
| Harborne et al. [71] | Lee Stocking Island (Bahamas) | MG, SG, RF | 17 |
| Harborne et al. [70] | San Salvador (Bahamas) | MG, SG, RF | 17 |
| Mumby et al. [16] | Belize | MG, SG, RF | 17 |
| Huijbers et al. [72] | Bermuda | MG, SG, RF | 17 |
| Nagelkerken unpubl. data* | Curaçao | MG, SG, RF | 17 |
| Sheridan [73] | Florida (USA) | MG, SG | 2 |
| Nagelkerken et al. [29] | Grand Cayman | MG, SG, RF | 17 |
| Harborne et al. [70] | Turks and Caicos Islands | MG, SG, RF | 17 |
|  |  |  |  |
|  | **Indo-Pacific** |  |  |
| Olds et al. [48,74] | Moreton Bay (Australia) | MG, SG, RF | 9 |
| Olds et al. [48] | Palm Islands (Australia) | MG, RF | 9 |
| Unsworth et al, [75] | Wakatobi, Sulawesi (Indonesia) | MG, SG | 4 |
| Nakamura et al. [41] | Ryukyu Islands (Japan) | SG, RF | 2 |
| Olds et al. [48,66] | Solomon Islands | MG, SG, RF | 9 |
| Kimirei et al. [7] | Kunduchi (Tanzania) | MG, SG, RF | 4 |
| Dorenbosch et al. [9] | Mafia (Tanzania) | MG, SG, RF | 21 |
| Kimirei et al. [7] | Mbegani (Tanzania) | MG, SG, RF | 4 |
| Dorenbosch et al. [9] | Pemba (Tanzania) | MG, RF | 21 |
| Dorenbosch et al. [9] | Zanzibar (Tanzania) | MG, SG, RF | 21 |
