# Supplementary material for: Mangrove Habitat Use by Juvenile Reef Fish: Meta-Analysis Reveals that Tidal Regime Matters More than Biogeographic Region
Source: PLoS One. 2014 Dec 31;9(12):e114715. doi: 10.1371/journal.pone.0114715 (PMC4281128; doi:10.1371/journal.pone.0114715)
Supplement: S2 Table — Random-effects categorical model summary statistics to test for the effect of species identity within habitat comparisons. (DOCX) [file pone.0114715.s003.docx]

**Supplementary Material**

Table S2. Random-effect categorical model summary statistics to test for the effect of species identity within habitat comparisons for seagrass beds (SG), mangroves (MG), and coral reefs (RF). Values indicate heterogeneity explained by the model (*Q_M_* ), the residual error heterogeneity (*Q_E_*), total heterogeneity (*Q_T_*), degrees of freedom (df) and p-values from a Chi-square (***χ*²**) distribution. Significant p-values for *Q_E_* * indicates that other underlying factors apart from species identity may be play a role in explaining some of the variability observed for the mean effect sizes for fish densities.

| **Region** | **Comparison** | **Model** | ***df*** | ***Q*** | **p-value (*χ*²)** |  |
| --- | --- | --- | --- | --- | --- | --- |
| **Caribbean** | SG–MG | *Q_M_* | 16 | 54.2 | <0.001 |  |
|  |  | *Q_E_* | 102 | 103.56 | 0.438 |  |
|  |  | *Q_T_* | 118 | 157.77 | 0.009 |  |
|  |  |  |  |  |  |  |
|  | RF–SG | *Q_M_* | 16 | 42.6 | <0.001 |  |
|  |  | *Q_E_* | 75 | 113.64 | 0.003* |  |
|  |  | *Q_T_* | 91 | 156.24 | <0.001 |  |
|  |  |  |  |  |  |  |
|  | RF–MG | *Q_M_* | 15 | 32.5 | 0.006 |  |
|  |  | *Q_E_* | 91 | 99.5 | 0.254 |  |
|  |  | *Q_T_* | 106 | 132 | 0.044 |  |
|  |  |  |  |  |  |  |
| **Indo-Pacific** | SG–MG | *Q_M_* | 15 | 21.18 | 0.131 |  |
|  |  | *Q_E_* | 31 | 45.37 | 0.046* |  |
|  |  | *Q_T_* | 46 | 66.55 | 0.025 |  |
|  |  |  |  |  |  |  |
|  | RF–SG | *Q_M_* | 17 | 34.54 | 0.007 |  |
|  |  | *Q_E_* | 35 | 52.19 | 0.031* |  |
|  |  | *Q_T_* | 52 | 86.73 | 0.002 |  |
|  |  |  |  |  |  |  |
|  | RF–MG | *Q_M_* | 17 | 57.29 | <0.001 |  |
|  |  | *Q_E_* | 53 | 200.97 | <0.001* |  |
|  |  | *Q_T_* | 70 | 258.26 | <0.001 |  |
